# Extracellular Vesicles Derived from Early and Late Stage *Plasmodium falciparum*-Infected Red Blood Cells Contain Invasion-Associated Proteins

**DOI:** 10.3390/jcm11144250

**Published:** 2022-07-21

**Authors:** Sinmanus Vimonpatranon, Sittiruk Roytrakul, Narumon Phaonakrop, Kittima Lekmanee, Anyapat Atipimonpat, Narinee Srimark, Kasama Sukapirom, Kesinee Chotivanich, Ladawan Khowawisetsut, Kovit Pattanapanyasat

**Affiliations:** 1 Graduate Program in Immunology, Department of Immunology, Faculty of Medicine Siriraj Hospital, Mahidol University, Bangkok 10700, Thailand; sinmanus.v@gmail.com; 2 Functional Ingredients and Food Innovation Research Group, National Center for Genetic Engineering and Biotechnology, Pathumthani 12120, Thailand; sittiruk@biotec.or.th (S.R.); narumon.pha@biotec.or.th (N.P.); 3 Siriraj Center of Research Excellence for Microparticle and Exosome in Diseases, Research Department, Faculty of Medicine Siriraj Hospital, Mahidol University, Bangkok 10700, Thailand; kittima.lem@gmail.com (K.L.); narinee.sri@mahidol.ac.th (N.S.); kasama.suk@mahidol.ac.th (K.S.); 4 Department of Biochemistry, Faculty of Medical Science, Naresuan University, Phitsanulok 65000, Thailand; anyapata@nu.ac.th; 5 Department of Clinical Tropical Medicine, Faculty of Tropical Medicine, Mahidol University, Bangkok 10400, Thailand; kesinee.cho@mahidol.ac.th; 6 Department of Parasitology, Faculty of Medicine Siriraj Hospital, Mahidol University, Bangkok 10700, Thailand

**Keywords:** *Plasmodium falciparum*, extracellular vesicles, malaria, proteomics, invasion

## Abstract

In infectious diseases, extracellular vesicles (EVs) released from a pathogen or pathogen-infected cells can transfer pathogen-derived biomolecules, especially proteins, to target cells and consequently regulate these target cells. For example, malaria is an important tropical infectious disease caused by *Plasmodium* spp. Previous studies have identified the roles of *Plasmodium falciparum*-infected red blood cell-derived EVs (*Pf*-EVs) in the pathogenesis, activation, and modulation of host immune responses. This study investigated the proteomic profiles of *Pf*-EVs isolated from four *P*. *falciparum* strains. We also compared the proteomes of EVs from (i) different EV types (microvesicles and exosomes) and (ii) different parasite growth stages (early- and late-stage). The proteomic analyses revealed that the human proteins carried in the *Pf*-EVs were specific to the type of *Pf*-EVs. By contrast, most of the *P. falciparum* proteins carried in *Pf*-EVs were common across all types of *Pf*-EVs. As the proteomics results revealed that *Pf*-EVs contained invasion-associated proteins, the effect of *Pf*-EVs on parasite invasion was also investigated. Surprisingly, the attenuation of parasite invasion efficiency was found with the addition of *Pf*-MVs. Moreover, this effect was markedly increased in culture-adapted isolates compared with laboratory reference strains. Our evidence supports the concept that *Pf*-EVs play a role in quorum sensing, which leads to parasite growth-density regulation.

## 1. Introduction

Malaria is an important tropical infectious disease leading to an estimated 240 million malaria cases in 2020 in 85 malaria-endemic countries [1]. The disease is caused by an apicomplexan protozoan, *Plasmodium* spp., transmitted to humans by the bite of female *Anopheles* mosquitos. Among five *Plasmodium* spp. that cause malaria in humans, *P. falciparum* is the most important because it is associated with severe syndromes and high mortality. The life cycle of *P. falciparum* in humans includes both the exo- and intraerythrocytic cycles in which the parasite grows and develops in hepatocytes or red blood cells (RBCs), respectively. During intraerythrocytic growth and development, the merozoites released from hepatocytes infect RBCs and asexually develop inside a parasitophorous vacuole from the ring stage to the trophozoite stage and schizont stage (containing many merozoites). At the end of schizogony, around 48 h after the invasion (for *P. falciparum*), infected RBCs rupture, and merozoites egress and, in turn, invade new RBCs, with the cycle repeating.

To infect the RBCs, the merozoite invasion progresses through a multi-step process consisting of initial attachment, apical reorientation, merozoite internalization, and parasitophorous vacuole membrane formation. Many ligand-receptor interactions between *P. falciparum* proteins take place on the merozoite surface and RBC receptors. In addition, signaling transduction-associated proteins sequentially participate in this process, facilitating parasite invasion. The list of essential merozoite proteins for the invasion process is extensive, including merozoite surface proteins (MSPs), erythrocyte-binding-like (EBL) and reticulocyte-binding-like (RBL) families, erythrocyte-binding antigen (EBA) and Rh protein family, *P. falciparum* reticulocyte-binding homolog 5 (*Pf*Rh5)/PfRh5-interacting protein (*Pf*Ripr)/cysteine-rich protective antigen (CyRPA) complex, rhoptry neck (RON) proteins, apical membrane antigen-1 (AMA-1), glycosylphosphatidylinositol-anchored micronemal antigen (GAMA), S-antigen, glutamate-rich protein (GLURP), serine-repeat antigen protein (SERA), claudin-like apicomplexan microneme protein (CLAMP), subtilisin-like serine protease (SUB), calcium-dependent protein kinases (CDPKs), glideosome-associated proteins (GAPs), myosin A, profilin, and cofilin [2,3,4,5,6,7,8,9]. Alterations in the function of these proteins by knockout gene expression or blocking (with inhibitors or antibodies) affect merozoite invasion efficiency and may inhibit parasite invasion [10,11].

During the intraerythrocytic growth cycle of *Plasmodium* spp., extracellular vesicles (EVs), which are nano-sized membrane-bound vesicles, are released from both uninfected and infected red blood cells (iRBCs) [12]. The EVs can be classified as an ectosome (microvesicles (MV) or microparticle (MP)) or exosome (Exo), based on their biogenesis and physical characteristics. These EVs function as mediators of intercellular communication via their cargos of biomolecules (proteins, lipids, metabolites, and genetic materials) from originated cells to recipient cells. EVs have also been reported to have essential roles in signal transduction, transcriptional regulation, and inflammatory reactions during the immune response in both pro- and anti-inflammatory phenomena [13,14,15,16]. The biological significance of EVs has been increasingly demonstrated across a broad range of pathologies, where their elevated levels in patient plasma are indicative of cellular stress and disease severity [17,18].

For more than two decades, EVs have been studied, and their roles in malaria infection have been shown to include regulating parasite growth, driving malaria pathogenesis, and modulating host immune responses. In experimental rodent malaria models, levels of MPs are high in the plasma of mice with cerebral malaria [19]. iRBC-derived MPs from the plasma of *Plasmodium berghei* strain ANKA (*Pb*A)-infected mice induce proinflammatory responses in macrophages resulting in systemic inflammation [20]. In addition, the adoptive transfer of MPs from *Pb*A-infected mice leads to localized MPs in cerebral microvessels of *Pb*A-infected recipient mice [19]. The decrease in MP production in knockout mice or with pharmacological inhibition protects these mice from cerebral malaria [21,22]. Similarly, EV levels are increased and associated with disease severity in malaria-infected patients [23,24]. Platelets and RBCs are major EV cell sources in patient plasma [24]. From in vitro experiments, *Pf*-EVs are internalized by monocytes and macrophages, deliver biomolecular cargos into the cytosol, and elicit innate immune cytokine responses [25,26,27]. Moreover, the internalization of *Pf*-EVs modulates endothelial cell barriers, including adherence-related proteins that lead to vascular dysfunction and support cytoadherence of iRBCs [28].

Previous studies indicate the role of *Pf*-EVs in parasite-parasite communication, which mediate the horizontal transfer of nucleic acids to regulate the formation of gametocytes (the sexual stage responsible for malaria transmission) [25,29]. Moreover, *Pf*-EVs derived from the cultures of low and high parasitemia carry *P. falciparum* lactate dehydrogenase (*Pf*LDH) in differential levels; only *Pf*-EVs derived from high parasite density regulate the parasite population by apoptosis induction [30]. This evidence implies the functional mechanism of *Pf*-EVs in a quorum sensing-like mechanism in which a cell secretes a signaling quorum-sensing molecule to communicate with other cells in response to the cell population density. As well-known studies reported in bacteria, the higher density of bacteria, higher level of the production and accumulation of quorum-sensing molecules. These molecules induce the positive feedback mechanism that allows communication between bacteria to regulate the gene expression involved in virulence, competition, pathogenicity, and resistance and to limit the population size [31]. The quorum sensing-like mechanism in protozoa has been proposed as the mechanism regulating the balance between virulence, survival in the host, and potential for transmission [32,33].

The proteomic profiles of malaria-derived EVs are now being studied. Sources of these EVs include plasma of *Plasmodium* spp.-infected patients, mice, and humanized mice, as well as from supernatants of *Plasmodium* spp. cultures [25,27,34]. Results show that these EVs contain both host and parasite proteins. Some have been identified as malaria antigens (MSP1, MSP3, MSP7, MSP9, serine-repeat antigen 1, and heat shock protein (HSP) 70), which might have potential for vaccine development [35]. Some might also be used as biomarkers of infection [34]. However, little information is available on the protein profiles of EVs derived from different *P. falciparum* strains. We hypothesized that the differences in *P. falciparum* strain virulence might be related to the EV’s selective and unique cell-specific proteins and thus their different disease outcomes. In this study, the proteomic profiles of EVs derived from different *P. falciparum* strains and growth stages were compared. We found that most virulence-associated proteins were highly conserved among *P.* *falciparum* strains. We further explored the effects of these *Pf*-EVs on invasion efficiency, growth, and development during intraerythrocytic schizogony.

## 2. Materials and Methods

### 2.1. Parasite Culture

Four *P. falciparum* parasite strains (3D7, NF54, TM01, and TM02) were grown in human RBCs in complete culture media (RPMI 1640 (Gibco; Grand Island, NY, USA) supplemented with 25 mM HEPES, 0.225% NaHCO_3_, 0.1 mM hypoxanthine, 25 g/mL gentamicin, and 5% AlbuMax II (Gibco; Auckland, New Zealand)) under an atmosphere of 5% O_2_, 5% CO_2_, and 90% N_2_. The culture media were changed daily. Parasite growth was monitored daily by Giemsa-stained thin blood smears. At a certain percentage of parasitemia, cultures were synchronized into the ring stage by resuspension of culture pellets in 5% D-sorbitol solution (Sigma-Aldrich; St. Louis, MO, USA) and incubated at 37 °C for 10 min, followed by centrifugation. The synchronized ring stage parasites were continuously re-cultured at 5% parasitemia and 5% hematocrit under an atmosphere of 5% O_2_, 5% CO_2_, and 90% N_2_ for 24 h. Culture supernatants were collected to isolate EVs of early-stage parasites (ring form to mid-trophozoite). New culture media were added and parasite cultures were continued for 24 h. During parasite growth beyond the mid-trophozoite stage, culture supernatants were collected to isolate EVs of late-stage parasites (mid-trophozoite to early-ring). These culture supernatants were centrifuged at 1500× *g* for 15 min to remove residual RBCs and cell debris and kept at −20 °C until used for EV isolation. This study was approved by the Siriraj Institutional Review Board of the Faculty of Medicine Siriraj Hospital, Mahidol University, Bangkok, Thailand (COA no Si 634/2019).

### 2.2. Extracellular Vesicle Isolation

The *Pf*-EVs were isolated from malarial culture supernatants by multi-step sequential centrifugation. Briefly, the 400 mL of cell-depleted culture supernatant was centrifuged at 13,000× *g* for 2 min and subsequently passed through a 1.2 µm syringe filter. The filtered culture supernatant was further centrifuged at 21,000× *g* (Thermo Scientific ^TM^ Sorvall RC-6 Plus superspeed centrifuge; Waltham, MA, USA), 4 °C for 70 min to collect the MV pellet. Then, the upper 90% of the supernatant was further passed through a 0.22 µm membrane filter and centrifuged at 110,000× *g* (Thermo Scientific ™ Sorvall WX80 ultracentrifuge), 4 °C for 90 min to collect the Exo pellet. Finally, these EV pellets were washed with sterile 0.22 µm-filtered phosphate-buffered saline (PBS) and stored at −80 °C until use. The EVs isolated from the supernatants of the cultures with early-stage development were designated as early-stage-derived MV (MV-E) and early-stage-derived Exo (Exo-E), while the EVs isolated from the culture supernatants of late-stage development were called late-stage-derived MV (MV-L) and late-stage-derived Exo (Exo-L), respectively. The control EVs were separated from the culture supernatant of 10 µM A23187-activated uninfected RBCs at 20% hematocrit for 24 h; culture supernatant was collected to isolate A23187-MV and A23187-Exo in the same manner as the *Pf*-EVs [36].

### 2.3. Transmission Electron Microscopy (TEM)

The morphology of *Pf*-EVs was visualized by TEM. First, the isolated *Pf*-EVs were fixed with 2% paraformaldehyde solution for 30 min. Next, the 200-mesh formvar/carbon-coated copper electron microscope (EM) grid was glow-discharged. The PFA-fixed *Pf*-EVs solution (3 µL) was loaded on the EM grid and incubated for 15 min. Then, the EM grid was stained with 50 µL of 2% uranyl acetate and incubated for 5–7 min. Finally, the excess solution was removed by filter paper and the EM grid was washed ten times with PBS and distilled water. The grid was imaged using an FEI Tecnai T12 Transmission Electron Microscope (Field Electron and Ion Co., Hillsboro, OR, USA) operated at 80 kV. Images were analyzed by DigitalMicrograph^®^ software (Gatan; Pleasanton, CA, USA).

### 2.4. Nanoparticle Tracking Analysis (NTA)

To characterize the size and particle concentration of *Pf*-EVs, the isolated EVs were diluted with sterile 0.22 µm-filtered PBS at the appropriate dilution to obtain the optimum measurement range. The diluted samples were injected into the Nanosight NS300 Analyzer (Malvern Pananalytical; Worcester, UK). Nanosight acquisition and analysis parameters were as follows: camera level: 14–15; detection threshold: 5; syringe pump speed: 50 µL/s; number of videos acquired per sample: 5; video duration: 60 s. Data analysis was performed using NTA software version 3.4 (Malvern Pananalytical; Worcester, UK). The system was washed with PBS between samples.

### 2.5. Western Blotting Analysis

The protein concentration of *Pf*-EVs was determined by Bradford protein assay (Bio-Rad Laboratories; Hercules, CA, USA) according to the manufacturer’s protocol. Isolated *Pf*-EVs (10 µg) were run on 12% SDS-polyacrylamide electrophoresis, and proteins were transferred to polyvinylidene difluoride membranes. These were blocked with non-fat dry milk in Tris-buffered saline containing 0.1% Tween20 (TBST), and the blots were probed with primary antibodies including β-actin (clone 8H10D10, Cell Signaling Technology^®^; Danvers, MA, USA), stomatin (clone E-5, Santa Cruz Biotechnology; Dallas, TX, USA), and flotillin-1 (clone C-2, Santa Cruz Biotechnology; Dallas, TX, USA), each diluted 1:400 in blocking buffer in TBST for 1 h. Then, the blots were washed with TBST and incubated with goat anti-mouse, PER-conjugated HRP (ImmunoTools GmbH; Friesoythe, Germany) at a dilution of 1:4000 for 1 h at room temperature. After washing, Clarity ™ Western ECL substrate was added (Bio-Rad Laboratories; Hercules, CA, USA); signal was detected by ImageQuant LAS 4010 (GE Healthcare; Boston, CA, USA).

### 2.6. Exosomal Protein Preparation

Total protein was isolated using 0.5% SDS solution and measured with Lowry assay using bovine serum albumin as the standard [37]. Protein samples (5 µg) were subjected to in-solution digestion. The samples were completely dissolved in 10 mM ammonium bicarbonate (AMBIC), then disulfide bonds were reduced with 5 mM dithiothreitol (DTT) in 10 mM AMBIC at 60 °C for 1 h; sulfhydryl groups were alkylated using 15 mM Iodoacetamide (IAA) in 10 mM AMBIC at room temperature for 45 min in the dark. For digestion, samples were mixed with 50 ng/µL of sequencing grade trypsin (1:20 ratio) (Promega; Madison, WI, USA) and incubated at 37 °C overnight. Before LC-MS/MS analysis, the digested samples were dried and protonated with 0.1% formic acid before injection into LC-MS/MS.

### 2.7. Liquid Chromatography-Tandem Mass Spectrometry (LC-MS/MS)

Tryptic peptide samples were injected in triplicate (5 µL each) into an HCT Ultra Ion Trap LC-MS System (Bruker Daltonics; Hamburg, Germany) which was coupled to a nanoLC system: UltiMate 3000 LC System (Thermo Fisher Scientific; Waltham, MA, USA) as well as an electrospray at the flow rate of 300 ոL/min to a nanocolumn (PepSwift monolithic column 100 mm internal diameter 50 mm). Each injection was spiked with 200 fmol of tryptic digested bovine serum albumin. Mobile phases of solvent A (0.1% formic acid) and solvent B (80% acetonitrile and 0.1% formic acid) were used to elute peptides, employing a linear gradient (10–70%) of solvent B at 0–13 min (the time-point of retention) followed by 90% solvent B at 13–15 min to remove all peptides in the column. A final elution of 10% solvent B at 15–20 min was carried out to remove any remaining salts.

### 2.8. Bioinformatics and Data Analysis

For protein quantitation, DeCyder MS Differential Analysis software (DeCyder MS, GE Healthcare; Boston, MA, USA) was used. Acquired LC-MS raw data were converted and the PepDetect module was used for automated peptide detection, charge state assignments, and quantitation based on peptide ion signal intensities in MS mode. The analyzed MS/MS data from DeCyder MS were submitted to the Mascot software version 2.2 (Matrix Science; London, UK) and searched against the Uniprot database for protein identification. The interrogation consisted of taxonomy (*Homo sapiens* or *Plasmodium falciparum*); enzyme (trypsin); variable modifications (carbamidomethyl, oxidation of methionine residues); mass values (monoisotopic); protein mass (unrestricted); peptide mass tolerance (1.2 Da); fragment mass tolerance (±0.6 Da); peptide charge state (1+, 2+ and 3+); max missed cleavages. Data normalization and quantification of differences in protein abundance between the control and treated samples were performed and visualized using MultiExperiment Viewer (MeV) software version 4.6.1 [38]. Briefly, peptide intensities from the LC-MS analyses were transformed and normalized using a mean central tendency procedure. Using statistical tests of variance of differences (ANOVA), the data sets were assessed for statistically significant amounts of protein (*p* < 0.05). Then, all differentially expressed proteins were analyzed for intersections among the different sample groups using jvenn [39]. Gene ontology annotation, including biological process, molecular function and cellular component, was performed using the PANTHER Classification System version 17.0 (http://www.pantherdb.org, accessed on 22 March 2022) [40]. The identified proteins were simultaneously submitted to Search Tool for Interacting Chemicals (STITCH) version 5.0 (http://stitch.embl.de, accessed on 5 May 2021) (European Molecular Biology Laboratory; Heidelberg, Germany) to search for linked cellular functions and other interactions between proteins and small molecules [41]. A heatmap analysis was generated using FunRich version 3.1.3 (http://www.funrich.org, accessed on 3 April 2022) [42].

### 2.9. Invasion and Growth Development Evaluation

Immature schizont parasites (42–44 h) were synchronized with the Percoll gradient solution [43]. In addition, the number of the schizont-infected and newly prepared RBCs were counted with BD Trucount^TM^ tubes (BD Biosciences; San Jose, CA, USA). Highly synchronized schizont-stage parasites (0.5% parasitemia in 1 × 10^7^ total RBCs) were seeded onto 96-well flat-bottom plates; each well contained a final culture volume of 100 µL. Isolated EVs were added into the cultures. Invasion efficiency was measured at 6 and 54 h post-coculturing by staining cultured RBCs with SYRB Green I nucleic acid staining dye; at least 50,000 RBCs were analyzed by BD^®^ LSR II Flow Cytometer (BD Biosciences; San Jose, CA, USA) [44]. Data were acquired using FACSDiva^TM^ software Version 7 (BD Biosciences; San Jose, CA, USA) and analyzed by FlowJo software Version 10.8.1 (Becton, Dickinson and Company; Ashland, OR, USA). The percentage parasitemia was determined by gating on SYRB Green I-positive RBCs. Each experiment was set up in triplicate wells, and the average parasitemia calculated.

### 2.10. Statistic Analysis

Data were analyzed using GraphPad Prism software version 9.3.1 (GraphPad; San Diego, CA, USA) and presented as mean ± SEM. The two-way ANOVA, followed by Tukey’s post-hoc test, was used to assess differences between more than two data groups. Differences were considered significant when the *p*-value was < 0.05.

## 3. Results

### 3.1. Characterization of Pf-Infected RBC-Derived EVs

**Figure 1:**
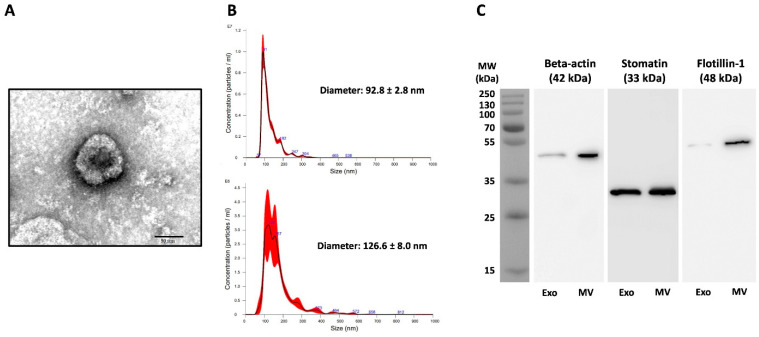
Characteristics of *Pf*-EVs isolated from supernatants of *P. falciparum* cultures. Exo, exosome; MV, microvesicle. (**A**) Representative TEM of *Pf*-Exo from *P. falciparum* 3D7. Image presents negatively stained *Pf*-Exo, imaged by TEM at amplification of 80,000X; the bar represents 50 nm. (**B**) Representative NTA results of *Pf*-Exo (**top**) and *Pf*-MV (**bottom**) from *P. falciparum* 3D7. (**C**) Representative Western blot of β-actin, stomatin, and flotillin-1 in *Pf*-Exo (**left lane**) and *Pf*-MV (**right lane**) from *P. falciparum* 3D7-infected RBCs.

The *Pf*-EVs and control A23187-EVs were isolated from culture supernatants by multi-step centrifugation, and their characteristics were analyzed. According to MISEV2018 criteria, the combination of images of single EVs and single-particle analysis should be performed to test the characteristics of EVs [45]. Thus, TEM and NTA were utilized to determine the characteristics of these *Pf*-EVs and control A23187-EVs. TEM showed the morphology of isolated *Pf*-Exo to be a cup-shaped membrane-bound vesicle approximately 100 nm in diameter (representative *Pf*-Exo; Figure 1A). By NTA, the average diameters of *Pf*-Exo and *Pf*-MV were 97.9 ± 8.1 nm and 159.7 ± 27.2 nm, respectively. A representative NTA showed that these sizes were 92.8 ± 2.8 nm and 126.6 ± 8.0 nm (Figure 1B). Western blot analysis was also performed to demonstrate the presence of EV markers in isolated *Pf*-EVs. β-actin and flotillin-1 were used as markers for cytosolic proteins and stomatin, an integral membrane protein of RBCs, was used as a marker of the plasma membrane of the originated cells. The results showed that both *Pf*-Exo (left lane) and *Pf*-MV (right lane) carried these three proteins, but at different levels. A representative Western blot analysis of *Pf*-MV and *Pf*-Exo isolated from 3D7 strain is shown (Figure 1C). The TEM, NTA and Western blots confirmed that *Pf*-EVs were successfully isolated from supernatants of malarial cultures.

### 3.2. Characterization of the Human Proteome in P. falciparum-Infected RBC-Derived EVs

To compare the proteomes found in *Pf*-EVs derived from different *P. falciparum* strains, the proteomic profiles of *Pf*-EVs from the different strains and developmental stages were identified with two databases: the human and *P. falciparum* protein databases.

Totals of 3717 and 3774 human proteins were found in MV and Exo isolated derived from supernatants of A23187-activated RBCs and *P. falciparum*-infected RBC cultures, respectively. Among these human proteins, EVs carried numerous RBC-specific proteins including hemoglobin subunit, acetylcholinesterase, excitatory amino acid transporter (EAAT), Band 3 anion transport protein, ankyrin, tropomodulin, CD44, CD47, glutathione S-transferase, thioredoxin reductase, heat shock protein 90-beta (HSP 90), carbonic anhydrase, and 14-3-3 protein [46,47]. Our isolated EVs contained several EV-specific proteins including AHCY, FLNA, ATP1A1, ENO1, EEF2, STOM, YWHAG, ALB, and RAB5A that are classified as Top100 proteins in the ExoCarta database and can be used as EV markers [48]. Thus, the EVs studied here contained both RBC-specific and EV-specific proteins.

**Figure 2:**
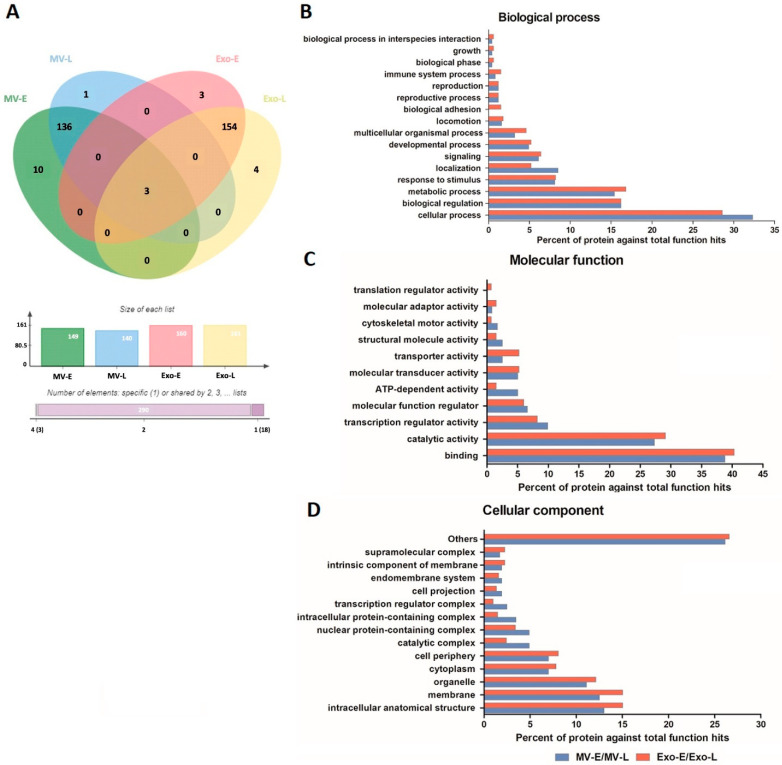
Human proteins in *Pf*-EVs of different type and developmental stage (MV-E, MV-L, Exo-E, and Exo-L). (**A**) Venn diagram showing the proteins differentially expressed in each type of *Pf*-EVs. The column graph presents the number of proteins in each type of EVs, and the bar graph indicates the number of proteins shared between types of EV. (**B**–**D**) GO annotation analyses of the common proteins in MV (MV-E/MV-L) and Exo (Exo-E/Exo-L); (**B**) biological process, (**C**) molecular function, and (**D**) cellular component.

When considering the human proteins present only in MV and Exo derived from *Pf*-infected RBC cultures, there were 150 proteins in *Pf*-MV and 164 proteins in *Pf*-Exo. Of these, only three proteins were expressed in both types of EVs. They were ATP-dependent RNA helicase DDX23 (Uniprot accession: Q9BUQ8), protein disulfide-isomerase A4 (Uniprot accession: P13667) and adenylate cyclase type 7 (Uniprot accession: P51828). Thus, there were 147 proteins found exclusively in *Pf*-MV and 161 proteins found only in *Pf*-Exo. In analyzing the proteins in *Pf*-EVs based on *P. falciparum* stage of development, 139 of the 150 proteins were found in both MV-E and MV-L. Ten proteins were uniquely found in MV-E, only one in MV-L. Among 164 human proteins in the Exo, 157 were found in both Exo-E and Exo-L. Three proteins were found uniquely in Exo-E, and four in Exo-L (Figure 2A). The human proteins detected uniquely in MV-E, MV-L, Exo-E, and Exo-L are listed in Table 1. These data suggested that the human proteins expressed in *Pf*-EVs were unique for the type of EVs but not the stage of *P. falciparum* development.

Next, the common human proteins expressed in *Pf*-MV and *Pf*-Exo were analyzed in terms of Gene Ontology (GO) annotation by PANTHER Classification System. The GO classification system revealed that the 138 proteins in *Pf*-MV (MV-E and MV-L) and 154 in *Pf*-Exo (Exo-E and Exo-L) could be classified based on functional properties, including those related to biological processes, molecular functions, and cellular components. The biological processes of proteins found commonly in both *Pf*-MV and *Pf*-Exo were the cellular process (GO:0009987), biological regulation (GO:0065007), and metabolic process (GO:0008152). The other functional classes (less than 10.0% each) identified were: response to stimulus (GO:0050896), localization (GO:0051179), signaling (GO:0023052), developmental process (GO:0032502), multicellular organismal process (GO:0032501), locomotion (GO:0040011), biological adhesion (GO:0022610), reproductive process (GO:0022414), reproduction (GO:0000003), immune system process (GO:0002376), growth (GO:0040007), biological phase (GO:0044848), and biological process involved in interspecies interaction between organisms (GO:0044419) (Figure 2B). Of molecular functions, the proteins in both *Pf*-MV and *Pf*-Exo were associated with binding (GO:0005488), catalytic activity (GO:0003824), transcription regulator activity (GO:0140110), molecular function regulator (GO:0098772), ATP-dependent activity (GO:0140657), molecular transducer activity (GO:0060089), transporter activity (GO:0005215), structural molecule activity (GO:0005198), cytoskeletal motor activity (GO:0003774), molecular adaptor activity (GO:0060090), and translation regulator activity (GO:0045182) (Figure 2C). In addition, the cellular components of proteins in both *Pf*-MV and *Pf*-Exo were involved in intracellular anatomical structure (GO:0005622), membrane (GO:0016020), organelle (GO:0043226), cell periphery (GO:0071944), cytoplasm (GO:0005737), catalytic complex (GO:1902494), nuclear protein-containing complex (GO:0140513), and intracellular protein-containing complex (GO:01400535) (Figure 2D).

**Figure 3:**
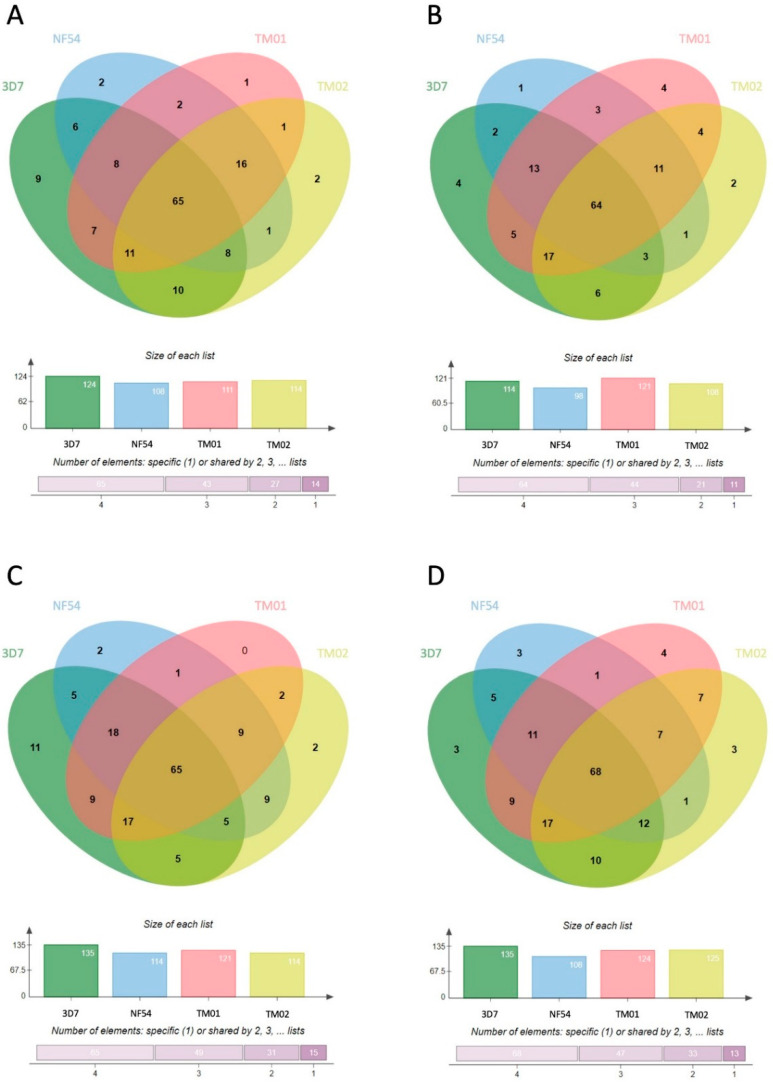
Venn diagrams showing the human proteins differentially found in each type of *Pf*-EVs from 4 *P. falciparum* strains (3D7, NF54, TM01, and TM02). (**A**) MV-E, n = 149; (**B**) MV-L, n = 140; (**C**) Exo-E, n = 160; (**D**) Exo-L, n = 161. The column graph presents the number of proteins in each strain of *P. falciparum,* and the bar graph indicates the cumulative number of proteins shared among the *P. falciparum* strains.

Comparison of specific proteins carried in each EV type from different *P. falciparum* strains showed that at least 40% of human proteins were found in EVs from all parasite strains. These common proteins made up 43.6% in MV-E, 45.7% in MV-L, 40.6% in Exo-E, and 42.2% in Exo-L. Less than 10% of these proteins were unique to a parasite strain in each EV group (Figure 3). These data suggested that many human proteins carried in *Pf*-EVs were conserved among different strains. However, the relative expression levels of these human proteins in *Pf*-EVs varied with parasite strain and stage. The heatmaps of unique human proteins carried in *Pf*-MV and *Pf*-Exo are shown in Figures S1 and S2, respectively.

### 3.3. Characterization of the P. falciparum Proteome in Pf-EVs

**Figure 4:**
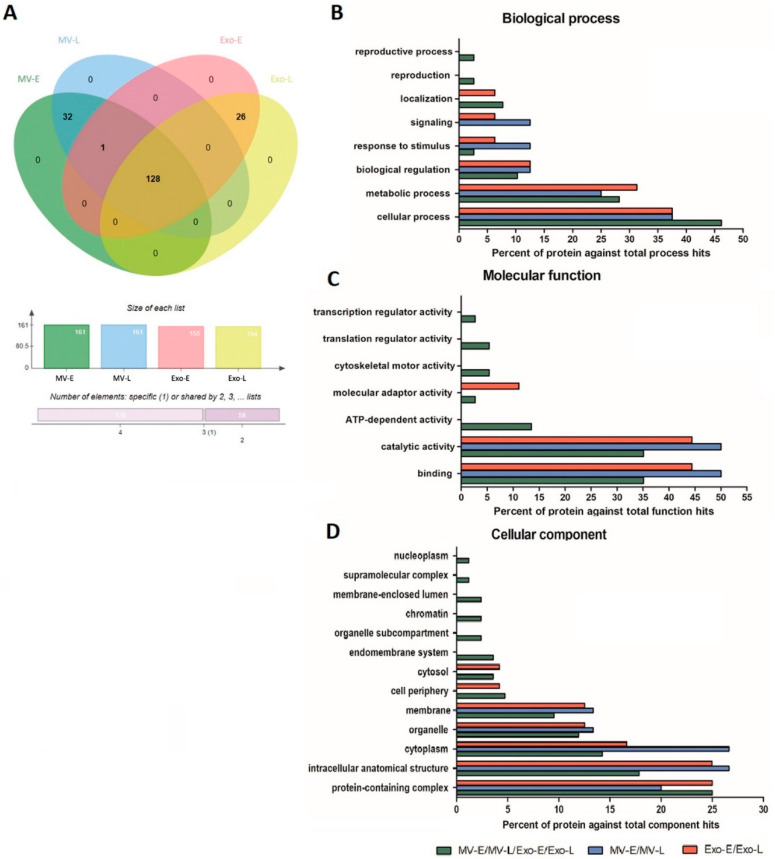
*P. falciparum* proteins in different types of *Pf*-EVs and stages of development (MV-E, MV-L, Exo-E, and Exo-L). (**A**) Venn diagram showed the proteins differentially expressed in each type of *Pf*-EVs. The column graph presents the number of proteins in each type of EVs, and the bar graph indicates the number of proteins shared between the type of EVs. (B–D) GO annotation analysis of the proteins common to EVs (MV-E/MV-L/Exo-E/Exo-L), MV (MV-E/MV-L), and Exo (Exo-E/Exo-L); (**B**) biological process, (**C**) molecular function, and (**D**) cellular component.

In contrast to human protein profiles, a Venn diagram analysis showed that the *P. falciparum* proteins in *Pf*-EVs can be divided into three subgroups: (i) proteins common to all *Pf*-EVs types (MV-E, MV-L, Exo-E, and Exo-L; 128 proteins), (ii) proteins expressed only in *Pf*-MV (MV-E and MV-L; 32 proteins), and (iii) proteins expressed only in *Pf*-Exo (Exo-E and Exo-L; 26 proteins) (Figure 4A). The *P. falciparum* proteins in each group were further grouped in terms of GO classification using PANTHER Classification System. The biological processes analysis showed that most identified proteins were involved in cellular processes (GO:0009987), followed by the metabolic processes (GO:0008152), biological regulation (GO:0065007), and response to stimulus (GO:0050896) (Figure 4B). The molecular functions analysis showed that identified proteins were mainly involved in binding (GO:0005488) and catalytic activity (GO:0003824) (Figure 4C). The cellular components analysis showed that identified proteins in *Pf*-EVs were involved in the protein-containing complex (GO:0032991), intracellular anatomical structure (GO:0005622), cytoplasm (GO:0005737), organelle (GO:0043226), and membrane (GO:0016020) (Figure 4D).

To explore potential functional interactions among the *P. falciparum* proteins in *Pf*-EVs, a network was built based on the STITCH 5 online database. An additional ten most-related proteins have expanded the network based on high-throughput text-mining [49]. Potential functional interactions among the *P. falciparum* proteins were found only in MVs (MV-E and MV-L), with 31 of 32 proteins in *Pf*-MV matching proteins in the database. Functional enrichment of the KEGG pathway identified the mismatch repair (pathway ID 03430, FDR = 0.00129), glutathione metabolism (pathway ID 00480, FDR = 0.0045), DNA replication (pathway ID 03030, FDR = 0.0045), nucleotide excision repair (pathway ID 03420, FDR = 0.0045), glycolysis/gluconeogenesis (pathway ID 00010, FDR = 0.0159), purine metabolism (pathway ID 00230, FDR = 0.0169), and metabolic pathways (pathway ID 01100, FDR = 0.0356). In *Pf*-Exo, 24 of 26 proteins matched with proteins in the database, and the functional enrichment of the KEGG pathway identified ribosome (pathway ID 03010, FDR = 2.72 × 10^−26^)

**Figure 5:**
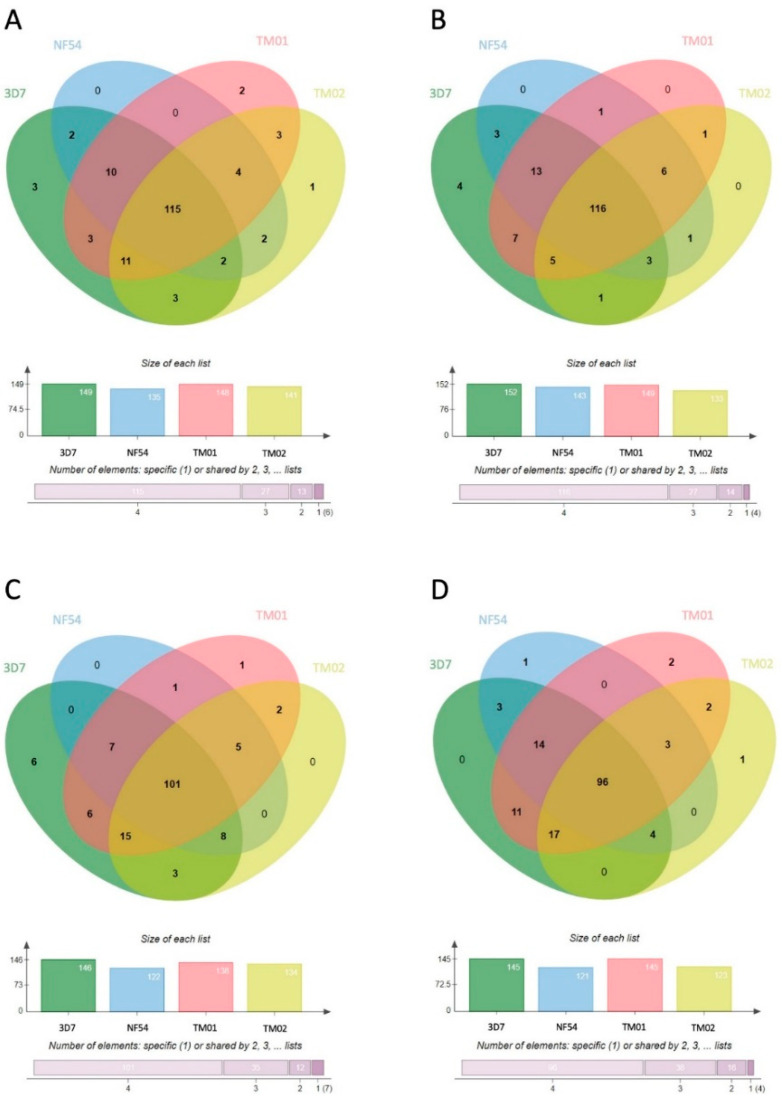
Venn diagrams showing the *P. falciparum* proteins differentially expressed in each type of *Pf*-EVs from 4 *P. falciparum* strains (3D7, NF54, TM01, and TM02). (**A**) MV-E, n = 161; (**B**) MV-L, n = 161; (**C**) Exo-E, n = 155; (**D**) Exo-L, n = 154. The column graphs present the number of proteins from each strain of *P. falciparum*, and the bar graphs indicate the numbers of proteins shared by *P. falciparum* strains.

In further evaluation of the protein diversity in EVs belonging to each *P. falciparum* strain, 71.4%, 72%, 65.1%, and 62.3% of proteins shared by all four strains were found in MV-E, MV-L, Exo-E, and Exo-L, respectively (Figure 5). These data suggested that the *P. falciparum* proteome in *Pf*-EVs was highly conserved across strains and that these percentages were higher than those belonging to the human proteome. Heatmap analyses comparing levels of *P. falciparum* proteins in each *Pf*-EVs type from the four *P. falciparum* strains are shown in Figure S3.

### 3.4. Pf-EVs Contain Parasite-Derived Proteins That Are Associated with Parasite Invasion

**Figure 6:**
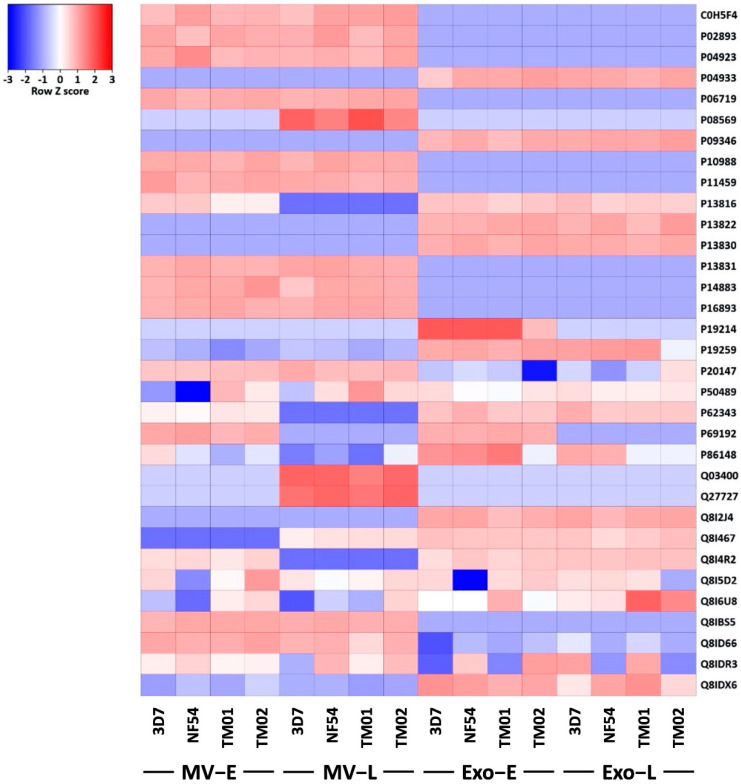
Heatmap of differential expression levels of invasion associated *P. falciparum* proteins detected in *Pf*-EVs. Side bar indicates differential protein expression between each sample (blue: decrease, red: increase). Rows: proteins; columns: samples.

Among *P. falciparum* proteins found in *Pf*-EVs, many were major virulence proteins which play an essential role in RBC invasion and erythrocytic schizogony, such as AMA-1, reticulocyte binding protein, MSP-1, and EBA-175. Potential invasion-associated proteins found in *Pf*-EVs are listed in Table 2. Furthermore, a heatmap analysis helped distinguish these proteins by *Pf*-EV type and parasite strain (Figure 6). These data persuaded us to explore the relationship of these *Pf*-EVs to invasion efficiency and parasite development during the intraerythrocytic schizogony of each *P. falciparum* strain.

### 3.5. Pf-EVs in Invasion Efficiency and Growth Development

The following experiment explored whether the *Pf*-EVs carrying invasion-associated *P. falciparum* proteins affected the merozoite RBC invasion process during the parasite’s life cycle. We added either MV (A23187-MV, *Pf*-MV-E and *Pf*-MV-L) or Exo (A23187-Exo, *Pf*-Exo-E and *Pf-*Exo-L) at 1 × 10^9^ particles into cultures containing 1 × 10^7^ RBCs. The cultures began with 0.5% parasitemia, and after two erythrocytic cycles were re-measured.

**Figure 7:**
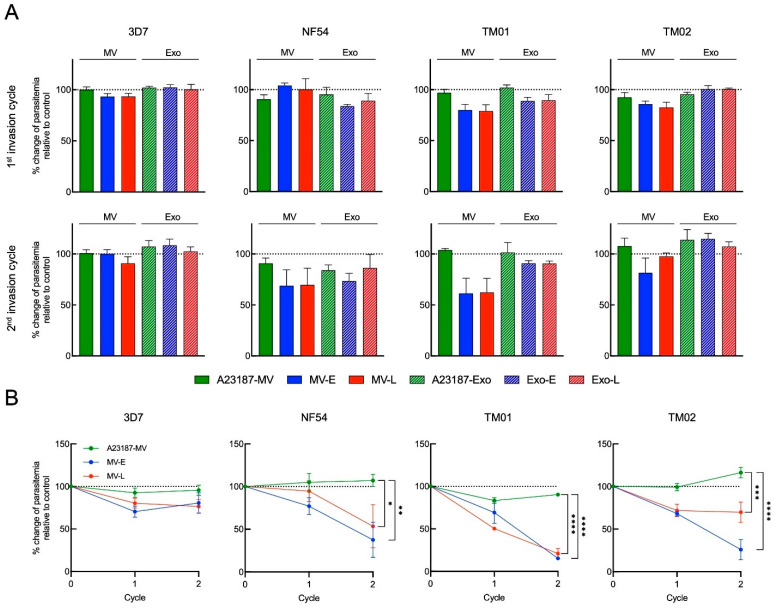
The effect of MV-E, MV-L, Exo-E and Exo-L treatment in *P. falciparum* invasion. (**A**) The percent change of parasitemia in the presence of 1 × 10^9^ EVs relative to control culturing without EVs in *P. falciparum* 3D7, NF54, TM01 and TM02, as indicated, at 6 h post-co-culture (**top row, first invasion cycle**) and 54 h post-co-culture (**bottom row, second invasion cycle**). (**B**) Line graphs represent percent change of parasitemia in the presence of 5 × 10^9^ *Pf*-MV relative to control culturing without MV over two invasion cycles of 3D7, NF54, TM01 and TM02, as indicated. Percent change of parasitemia at second invasion cycle were compared between control culturing without MV and MV treatment group using a two-way ANOVA, followed by Tukey’s post-hoc test (*: *p* < 0.05, **: *p* < 0.01, ***: *p* < 0.001, ****: *p* < 0.0001).

The first invasion cycle was determined at 6 h post co-incubation or after schizont rupture. The co-incubation then was continued over 48 h for second schizont rupture and was determined percentage of parasitemia as the second invasion cycle. The percent change of parasitemia was calculated relatively to control which was referred to the condition without the addition of EVs. In the first invasion cycle post-coincubation, the percentages of changes in parasitemia of control EVs (A23187-MV and A23187-Exo) from four strains were varied from 90.58 ± 4.38 to 102.04 ± 2.55, while those of *Pf*-MV coculture ranged from 80.15 ± 5.51 to 104.20 ± 2.38 and those of *Pf*-Exo coculture ranged from 83.90 ± 1.67 to 102.33 ± 2.54. Thus, the data seem to suggest that the effect of *Pf*-EVs on the invasion process was not clearly demonstrated at this time point. By contrast, in comparison with A23187-MV in the second invasion cycle, we observed a slight reduction in percent change in parasitemia with *Pf*-MV derived from NF54, TM01, and TM02 strains; *Pf*-Exo did not alter the culture parasitemia (Figure 7A).

To clarify the effect of *Pf*-MV on parasite invasion and growth, we increased the number of *Pf*-MV particles five-fold added to the cultures. As a result, there were short-term effects on *P. falciparum* development. However, when the co-cultures were observed through the second invasion cycle, significant reductions in parasite populations in cultures were seen with both MV-E and MV-L derived from NF54, TM01, and TM02 strains, but not from the 3D7 strain (Figure 7B). This finding suggested that EVs, especially *Pf*-MV, secreted from iRBCs moderate parasite growth of many *P. falciparum* strains.

## 4. Discussion

Like many other pathogens, EVs secreted from *Plasmodium* malaria play a role in disease complications. Indeed, elevated levels of circulating EVs have been detected in plasma of malaria-infected patients and correlated with severity of the disease [12,21]. Recently, RBC EVs derived from *P. falciparum* culture supernatants were widely investigated. In vitro studies demonstrated the role of RBC EVs in intercellular communication between parasite populations. Parasite genetic material is transferred via RBC EVs and internalized into iRBCs, influencing the transmission stage of parasites [25,29]. Obviously, parasite growth in vitro has limited maximum growth capacity. RBC EVs are reported to have a key role in the control of parasite density [30]. However, the way in which EVs impact in vitro cultures may reveal interesting mechanisms. For example, the priming of naïve RBCs with EVs supports parasite growth by altering membrane stiffness [50]. Thus, the involvement of EVs in intercellular parasite communication (parasite–parasite and host–parasite) is complex and needs to be better elucidated [51,52,53].

EVs play a key role in intercellular communication by transferring bioactive molecules such as proteins and genetic materials from parasites to host cells and parasites to parasites, thereby modifying the properties of recipient cells [25,27,34,50,54,55,56]. EVs in plasma have several cellular origins. EVs in supernatants of *P. falciparum*-RBC cultures are less numerous than in plasma of malaria patients. In this study, *Pf*-EVs were derived from the cultures of four *P. falciparum* strains, 3D7, NF54, TM01, and TM02. *P. falciparum* NF54 is a strain isolated from a patient with uncomplicated malaria in the Netherlands, but the origin of the parasite is thought to be in Africa [57,58]. *P. falciparum* 3D7 is one of the most studied strains of *P. falciparum* in research laboratories and is a clone from *P. falciparum* strain NF54. Although the NF54 and 3D7 are assumed to be genetically identical, phenotypic differences when grown in culture are reported [59]. The *P. falciparum* strains TM01 and TM02 were culture-adapted isolates which collected from uncomplicated and severe falciparum malaria patients in Thailand, respectively. In this study, EVs were termed as MV or Exo based on differential centrifugation at 21,000× *g* and 110,000× *g*, respectively. The average diameter of MV was 159.7 ± 27.2 nm, and the average diameter of Exo was 97.9 ± 8.1 nm. We admit that our separation protocol may not be sufficient to isolate *Pf*-MV and *Pf*-Exo as an overlap of the size ranges of both EVs was observed.

To determine protein content during distinct stages of development, parasites were synchronized and RBC culture supernatants were harvested after 24 and 48 h to acquire early and late stages, respectively. From the comparative proteomic profiling, both host and parasite proteins were identified in EVs. Among human proteins, the majority involved RBC membrane and cytosolic proteins such as hemoglobin, band 3, band 7 (stomatin), spectrin, CD47, CD55, acetylcholinesterase, and carbonic anhydrase, which are known to be enriched in RBC EVs [60,61,62]. When considering the human proteins found only in *Pf*-EVs (not in A23187-EVs), there were 150 proteins in *Pf*-MV and 164 proteins in *Pf*-Exo. Of these, only three proteins were expressed in both types of EVs. Considering the parasite protein profiles in early- and late-stage EVs, less than ten proteins were unique to one stage or the other (Table 1). Thus, human proteins expressed in *Pf*-EVs were unique for the type of EVs but not the stage of parasite and were common across strains. In contrast to human proteins, *P. falciparum* proteins were most often common to all *Pf*-EVs types (n = 128 proteins), but 32 were found only in *Pf*-MV and 26 only in *Pf*-Exo. Moreover, 62–72% of proteins were associated with all four strains.

Interestingly, *P. falciparum* protein profiles revealed several invasion-associated proteins in *Pf*-EVs. For example, MSP-1 (an essential protein in the parasite’s initial contact), proteins involved in the apical orientation step (EBA-175, PfRh1, PfRh2a, PfRh2b, and PfRh3), and AMA1 (a crucial protein in tight junction formation) were all found. Moreover, these parasite proteins are common in RBC EVs derived from *P. falciparum* culture supernatants [25,27,30,50,56,63]. Other interesting parasite proteins that were identified by proteomics include two proteins linked to the iRBC surface membrane, RESA and KAHRP. While these *P. falciparum* proteins were present in *Pf*-EVs from all four strains, the relative expression levels were observed. For instance, myosin-A, GBP-130, and AMA1 were less frequent in *Pf*-MV from 3D7 in comparison with other strains.

Focusing on the influence of EVs on parasite growth development, there have been interesting approaches to study this aspect. Mantel et al. profiled EV products and identified abundant RBC lipid raft proteins, RBC membrane proteins and others involved in parasite invasion of RBCs [25]. Their approach was to treat ring stage parasites with RBC EVs and investigate parasitemia after 48 h. However, they did not observe significant alterations in growth rates. In contrast to this finding, we showed that *Pf*-EVs, in particular, *Pf*-MVs from all strains, had more effect in terms of attenuating parasite invasion efficiency than did *Pf*-Exo, and the effects of *Pf*-MVs were significant only at high dose. Our study is in line with a report by Correa et al., who modified the approach by adding EVs isolated from high parasitemia cultures into those with mature trophozoites; they observed significant reductions in growth after 24 h [30]. Moreover, they identified *Pf*-LDH as an inducer of apoptosis and parasite density control. Recently, Dekel et al. modified the experiment again by priming naïve uninfected-RBCs (uRBCs) with *Pf*-EVs for 18 h and then introduced them to late-stage parasites [50]. After 12 h, they found an increase in parasite levels in the EV pretreatment cultures. They identified 20S proteasome as a key feature in altering RBC membrane stiffness and so facilitating parasite growth. In consistent with this report, we found both human and parasite proteins except that we did not find proteasomes of parasite origin. These discrepancies in the protein contents could be partially ascribed to: (1) Different approach in treating uRBCs with *Pf*-EVs. In our study, uRBCs were exposed to both *Pf*-EVs and parasites at the same time during malaria cultures without pretreatment with *Pf*-EVs. (2) Different stages of *Pf*-EVs that were used to treat the uRBCs. Unlike Dekel et al., who used late-stage *Pf*-EVs [50], our experiment used both early- and late stage-derived EVs. It is reasoned that EVs at different stages of parasite development exploit distinct selective sorting mechanisms for proteins and their mRNAs. Cumulative evidence suggests the existence of regulators that control the sorting of protein/mRNA cargo into EVs and their release by utilizing parasite own protein network [26,29,50,64]. While further study is needed to understand the complex molecular nature of such differences in proteomes, we suggest that such differences might be associated with distinct cargo sorting at each stage of parasite development which might have a crucial role in parasite–host communication.

Because certain *P. falciparum* strains are considered more virulent than others, and parasites causing severe malaria have greater invasion potential with less selective and multiplied more at high parasitemia than those of uncomplicated malaria [50,65,66]. An analogy with this notion would suggest that EVs from virulent and non-virulent malaria parasites may have different multiplication rates and invasiveness when invading host RBCs. In an effort to better understand the effect of *Pf*-EVs on the invasion efficiency and growth development during intraerythrocytic schizogony of each *P. falciparum* strain, in this study, we determined the effects of EVs both immediately after schizogony and then again after a second invasion cycle. Surprisingly, *Pf*-MVs from all strains had more of an effect attenuating parasite invasion efficiency than *Pf*-Exo. However, the effects of *Pf*-MVs were significant only at a high dose. Interestingly, no significant difference in parasite growth was observed when EVs isolated from uncomplicated (TM01) and severe (TM02) falciparum malaria were used to treat uRBCs. It should be emphasized here that the parasite growth in our study has limitations that must be considered; since our approach used an in vitro model, it was limited by the lack of a host immune response since RBCs were the only source of EVs. These limitations might explain the similar findings among strains that differed in virulence. Though the protein contents present in *Pf*-EVs were highly conserved among all four *P*. *falciparum* strains, the effects of *Pf*-MV were markedly different in culture-adapted isolates versus laboratory reference strains. Long-term *P*. *falciparum* laboratory strains, such as 3D7, have been adapted to easily maintain the culture, whereas more recently acquired *P*. *falciparum* isolates might be more susceptible to intercellular communication. However, as the effect of EVs in invasion activities is from the whole EVs activity, the results cannot indicate the exact potential essential proteins in the process. In addition, because we did not include the drug-resistant *P. falciparum* strains in the study, questions about the differences in EVs from these strains still need further investigation.

This finding provides some knowledge to better understand the role of *Pf*-EVs in regulating malaria parasite growth. Furthermore, as *Pf*-EVs act as cargo carrying the quorum-sensing molecules with invasion inhibitory activity, identifying potential quorum-sensing molecules in *Pf*-EVs might benefit the new therapeutic approach and management during malaria infection. For example, administering the current antimalarial drugs combined with synthetic quorum-sensing molecules or bioengineered EVs carrying quorum-sensing molecules, these should synergize with each other in controlling the parasitemia in drug-resistance malaria strain. In addition, the EVs or quorum-sensing molecules might be used as a biomarker for cell-free diagnosis in the endemic malaria area, in which the mutant *P. falciparum* parasites lack histidine-rich protein and cannot be detected with the present rapid diagnostic tests.

## 5. Conclusions

This work investigated protein cargos in *Pf*-EVs generated from four strains of *P. falciparum* using LC-MS/MS analysis. Their protein profiles revealed human proteins related to RBC EV markers and highly conserved *P. falciparum* proteins associated with the RBC invasion process. Unexpectedly, *Pf*-MVs had the effect of attenuating, rather than supporting, parasite invasion efficiency. Our evidence supports the involvement of *Pf*-EVs in growth-density regulation via a quorum sensing-like mechanism, a process observed across strains of *P. falciparum*.

## Figures and Tables

**Table 1:** List of human proteins detected only in MV-E, MV-L, Exo-E, and Exo-L.

Uniprot Accession	Entry Name	Protein Name
**Proteins detected only in MV-** **E**		
O75140	DEPD5_HUMAN	DEPDC5-DEP domain containing 5
P50226	ST1A2_HUMAN	SULT1A2-sulfotransferase family, cytosolic, 1A, phenol-preferring, member 2
P56696	KCNQ4_HUMAN	KCNQ4-potassium voltage-gated channel, KQT-like subfamily, member 4
Q14C86	GAPD1_HUMAN	GAPVD1-GTPase activating protein and VPS9 domains 1
Q5U623	MCAF2_HUMAN	ATF7IP2-activating transcription factor 7 interacting protein 2
Q86YA3	ZGRF1_HUMAN	C4orf21-chromosome 4 open reading frame 21
Q8TD57	DYH3_HUMAN	DNAH3-dynein, axonemal, heavy chain 3
Q96QD9	UIF_HUMAN	FYTTD1-forty-two-three domain containing 1
Q9HC35	EMAL4_HUMAN	EML4-echinoderm microtubule associated protein like 4
Q9NP80	PLPL8_HUMAN	PNPLA8-patatin-like phospholipase domain containing 8
**Proteins detected only in MV-** **L**		
Q14692	BMS1_HUMAN	BMS1-BMS1 homolog, ribosome assembly protein
**Proteins detected only in Exo-** **E**		
Q13546	RIPK1_HUMAN	RIPK1-receptor (TNFRSF)-interacting serine-threonine kinase 1
Q15645	PCH2_HUMAN	TRIP13-thyroid hormone receptor interactor 13
Q86YN1	DOPP1_HUMAN	DOLPP1-dolichyl pyrophosphate phosphatase 1
**Proteins detected only in Exo-** **L**		
O60308	CE104_HUMAN	CEP104-centrosomal protein 104 kDa
Q09328	MGT5A_HUMAN	MGAT5-mannosyl (alpha-1,6-)-glycoprotein beta-1,6-N-acetyl-glucosaminyltransferase
Q8N1B4	VPS52_HUMAN	VPS52-vacuolar protein sorting 52 homolog (S. cerevisiae)
Q9Y613	FHOD1_HUMAN	FHOD1-formin homology 2 domain containing 1

**Table 2:** List of invasion-associated *P. falciparum* proteins detected in *Pf*-*EVs.*

Uniprot Accession	Entry Name	Protein Name	Function
C0H5F4	RBP2B_PLAF7	Reticulocyte binding protein 2 homolog b	Involved in reticulocyte adhesion
P02893	CSP_PLAFA	Circumsporozoite protein (CS)	Immunodominant surface antigen on the sporozoite
P04923	CRA_PLAFA	Circumsporozoite protein-related antigen (CRA)	Located within parasitophorous vacuole and associated with membranous structures in RBC cytoplasm
P04933	MSP1_PLAFW	Merozoite surface protein 1 (Merozoite surface antigens) (PMMSA) (p195)	Pathogenesis
P06719	KNOB_PLAFN	Knob-associated histidine-rich protein (KAHRP)	Mimic human histidine-rich glycoproteins to anchor host thrombospondin or a parasite analog in a binding complex with the endothelial cell receptor
P08569	MSP1_PLAFM	Merozoite surface protein 1 (Merozoite surface antigens) (PMMSA) (p190)	Pathogenesis
P09346	KNOB_PLAFG	Knob-associated histidine-rich protein (KAHRP) (KP)	Mimic human histidine-rich glycoproteins to anchor host thrombospondin or a parasite analog in a binding complex with the endothelial cell receptor
P10988	ACT1_PLAFO	Actin-1 (Actin I) (Pf-actin I)	Contribute to parasite gliding motility
P11459	RHOA_PLAFA	Rhoptry antigen protein (Fragment)	Participate in the invasion of RBCs by merozoites
P13816	GARP_PLAFF	Glutamic acid-rich protein	Enhance the adhesive properties of human RBC by engaging band 3 receptor
P13822	SANT_PLAFP	S-antigen protein (Fragment)	Soluble heat-stable proteins
P13830	RESA_PLAFF	Ring-infected erythrocyte surface antigen	Facilitate the invagination of the red cell membrane which is necessary for the formation of the parasitophorous vacuole.
P13831	RESA_PLAFN	Ring-infected erythrocyte surface antigen (Fragment)	
P14883	ACT2_PLAFO	Actin-2 (Actin II) (Pf-actin II)	Contribute to parasite gliding motility
P16893	TRAP_PLAFA	Thrombospondin-related anonymous protein	Cell adhesion
P19214	EBA1_PLAFC	Erythrocyte-binding antigen 175 (EBA-175)	Host cell surface receptor binding
P19259	PF12_PLAFA	Merozoite surface protein PF12	Cleaved from the surface during invasion
P20147	HSP90_PLAFP	Heat shock 90 kDa protein homolog (Fragment)	Molecular chaperone
P50489	AMA1_PLAFC	Apical membrane antigen 1 (Merozoite surface antigen)	Involved in parasite invasion of erythrocytes
P62343	CDPK1_PLAFK	Calcium-dependent protein kinase 1 (EC 2.7.11.1) (PfCDPK1) (PfCPK)	Required for microneme secretion and thus merozoite egress from and invasion of host erythrocytes
P69192	SERA_PLAFG	Serine-repeat antigen protein (111 kDa antigen) (p126)	It may function at the RBC membrane, perhaps as a component that influences the invasion process
P86148	RBP1_PLAF7	Reticulocyte-binding protein PFD0110w	Involved in reticulocyte adhesion
Q03400	SANT_PLAF7	S-antigen protein	Soluble heat-stable proteins
Q27727	ENO_PLAFA	Enolase (EC 4.2.1.11) (2-phospho-D-glycerate hydrolyase) (2-phosphoglycerate dehydratase)	Catalytic activity
Q8I2J4	PROF_PLAF7	Profilin	Essential for the invasive blood stages of the parasite
Q8I467	CADF1_PLAF7	Cofilin/actin-depolymerizing factor homolog 1	Essential for erythrocytic schizogony
Q8I4R2	RBP3_PLAF7	Reticulocyte-binding protein 3	Involved in reticulocyte adhesion
Q8I5D2	ABRA_PLAF7	101 kDa malaria antigen (Acidic basic repeat antigen) (p101)	Located at the merozoite surface, within the parasitophorous vacuole of *P. falciparum*
Q8I6U8	GBP_PLAF7	Glycophorin-binding protein (GBP-130)	Involved in erythrocyte invasion
Q8IBS5	CDPK4_PLAF7	Calcium-dependent protein kinase 4 (EC 2.7.11.1)	Host RBCs and hepatocytes infection cycles, sexual reproduction and mosquito transmission of the parasite
Q8ID66	PF92_PLAF7	Merozoite surface protein PF92	Cys-rich surface protein; binds factor H andinvolved in complement evasion
Q8IDR3	MYOA_PLAF7	Myosin-A (PfM-A)	Actin-based motor molecules with ATPase activity which critical for *P. falciparum* RBC invasion
Q8IDX6	RBP2A_PLAF7	Reticulocyte-binding protein 2 homolog a	Involved in reticulocyte adhesion

## Data Availability

Not applicable.

## Supplementary Materials

The following supporting information can be downloaded at: https://www.mdpi.com/article/10.3390/jcm11144250/s1, Figure S1: Heatmap of unique human proteins carried in *Pf*-MV; Figure S2: Heatmap of unique human proteins carried in *Pf*-Exo; Figure S3: Heatmap analysis of differential expression levels of *P. falciparum* protein in each type of *Pf*-EVs from 4 *P. falciparum* strains

## References

[1] WHO (2021). World Malaria Report 2021.

[2] Volz J.C., Yap A., Sisquella X., Thompson J.K., Lim N.T., Whitehead L.W., Chen L., Lampe M., Tham W.H., Wilson D. (2016). Essential role of the PfRh5/PfRipr/CyRPA complex during *Plasmodium falciparum* invasion of erythrocytes. Cell Host Microbe.

[3] Kato K., Mayer D.C., Singh S., Reid M., Miller L.H. (2005). Domain III of *Plasmodium falciparum* apical membrane antigen 1 binds to the erythrocyte membrane protein Kx. Proc. Natl. Acad. Sci. USA.

[4] Treeck M., Zacherl S., Herrmann S., Cabrera A., Kono M., Struck N.S., Engelberg K., Haase S., Frischknecht F., Miura K. (2009). Functional analysis of the leading malaria vaccine candidate AMA-1 reveals an essential role for the cytoplasmic domain in the invasion process. PLoS Pathog..

[5] Lu J., Chu R., Yin Y., Yu H., Xu Q., Yang B., Sun Y., Song J., Wang Q., Xu J. (2022). Glycosylphosphatidylinositol-anchored micronemal antigen (GAMA) interacts with the band 3 receptor to promote erythrocyte invasion by malaria parasites. J. Biol. Chem..

[6] Schlott A.C., Knuepfer E., Green J.L., Hobson P., Borg A.J., Morales-Sanfrutos J., Perrin A.J., Maclachlan C., Collinson L.M., Snijders A.P. (2021). Inhibition of protein N-myristoylation blocks *Plasmodium falciparum* intraerythrocytic development, egress and invasion. PLoS Biol..

[7] Robert-Paganin J., Robblee J.P., Auguin D., Blake T.C.A., Bookwalter C.S., Krementsova E.B., Moussaoui D., Previs M.J., Jousset G., Baum J. (2019). Plasmodium myosin A drives parasite invasion by an atypical force generating mechanism. Nat. Commun..

[8] Patarroyo M.A., Molina-Franky J., Gomez M., Arevalo-Pinzon G., Patarroyo M.E. (2020). Hotspots in Plasmodium and RBC Receptor-Ligand Interactions: Key Pieces for Inhibiting Malarial Parasite Invasion. Int. J. Mol. Sci..

[9] Cowman A.F., Tonkin C.J., Tham W.H., Duraisingh M.T. (2017). The Molecular basis of erythrocyte invasion by malaria parasites. Cell Host Microbe.

[10] Lopaticki S., Maier A.G., Thompson J., Wilson D.W., Tham W.H., Triglia T., Gout A., Speed T.P., Beeson J.G., Healer J. (2011). Reticulocyte and erythrocyte binding-like proteins function cooperatively in invasion of human erythrocytes by malaria parasites. Infect. Immun..

[11] Williams A.R., Douglas A.D., Miura K., Illingworth J.J., Choudhary P., Murungi L.M., Furze J.M., Diouf A., Miotto O., Crosnier C. (2012). Enhancing blockade of *Plasmodium falciparum* erythrocyte invasion: Assessing combinations of antibodies against PfRH5 and other merozoite antigens. PLoS Pathog..

[12] Nantakomol D., Dondorp A.M., Krudsood S., Udomsangpetch R., Pattanapanyasat K., Combes V., Grau G.E., White N.J., Viriyavejakul P., Day N.P. (2011). Circulating red cell-derived microparticles in human malaria. J. Infect. Dis..

[13] Slomka A., Urban S.K., Lukacs-Kornek V., Zekanowska E., Kornek M. (2018). Large extracellular vesicles: Have we found the holy grail of inflammation?. Front. Immunol..

[14] Hezel M.E.V., Nieuwland R., Bruggen R.V., Juffermans N.P. (2017). The Ability of Extracellular vesicles to induce a pro-inflammatory host response. Int. J. Mol. Sci..

[15] Robbins P.D., Morelli A.E. (2014). Regulation of immune responses by extracellular vesicles. Nat. Rev. Immunol..

[16] Zhou X., Xie F., Wang L., Zhang L., Zhang S., Fang M., Zhou F. (2020). The function and clinical application of extracellular vesicles in innate immune regulation. Cell Mol. Immunol..

[17] Maas S.L.N., Breakefield X.O., Weaver A.M. (2017). Extracellular vesicles: Unique intercellular delivery vehicles. Trends Cell Biol..

[18] Julich H., Willms A., Lukacs-Kornek V., Kornek M. (2014). Extracellular vesicle profiling and their use as potential disease specific biomarker. Front. Immunol..

[19] El-Assaad F., Wheway J., Hunt N.H., Grau G.E., Combes V. (2014). Production, fate and pathogenicity of plasma microparticles in murine cerebral malaria. PLoS Pathog..

[20] Couper K.N., Barnes T., Hafalla J.C., Combes V., Ryffel B., Secher T., Grau G.E., Riley E.M., de Souza J.B. (2010). Parasite-derived plasma microparticles contribute significantly to malaria infection-induced inflammation through potent macrophage stimulation. PLoS Pathog..

[21] Combes V., Coltel N., Alibert M., van Eck M., Raymond C., Juhan-Vague I., Grau G.E., Chimini G. (2005). ABCA1 gene deletion protects against cerebral malaria: Potential pathogenic role of microparticles in neuropathology. Am. J. Pathol..

[22] Penet M.F., Abou-Hamdan M., Coltel N., Cornille E., Grau G.E., de Reggi M., Gharib B. (2008). Protection against cerebral malaria by the low-molecular-weight thiol pantethine. Proc. Natl. Acad. Sci. USA.

[23] Campos F.M., Franklin B.S., Teixeira-Carvalho A., Filho A.L., de Paula S.C., Fontes C.J., Brito C.F., Carvalho L.H. (2010). Augmented plasma microparticles during acute *Plasmodium vivax* infection. Malar. J..

[24] Pankoui Mfonkeu J.B., Gouado I., Fotso Kuate H., Zambou O., Amvam Zollo P.H., Grau G.E., Combes V. (2010). Elevated cell-specific microparticles are a biological marker for cerebral dysfunctions in human severe malaria. PLoS ONE.

[25] Mantel P.Y., Hoang A.N., Goldowitz I., Potashnikova D., Hamza B., Vorobjev I., Ghiran I., Toner M., Irimia D., Ivanov A.R. (2013). Malaria-infected erythrocyte-derived microvesicles mediate cellular communication within the parasite population and with the host immune system. Cell Host Microbe.

[26] Sisquella X., Ofir-Birin Y., Pimentel M.A., Cheng L., Abou Karam P., Sampaio N.G., Penington J.S., Connolly D., Giladi T., Scicluna B.J. (2017). Malaria parasite DNA-harbouring vesicles activate cytosolic immune sensors. Nat. Commun..

[27] Sampaio N.G., Emery S.J., Garnham A.L., Tan Q.Y., Sisquella X., Pimentel M.A., Jex A.R., Regev-Rudzki N., Schofield L., Eriksson E.M. (2018). Extracellular vesicles from early stage *Plasmodium falciparum*-infected red blood cells contain PfEMP1 and induce transcriptional changes in human monocytes. Cell Microbiol..

[28] Mantel P.Y., Hjelmqvist D., Walch M., Kharoubi-Hess S., Nilsson S., Ravel D., Ribeiro M., Gruring C., Ma S., Padmanabhan P. (2016). Infected erythrocyte-derived extracellular vesicles alter vascular function via regulatory Ago2-miRNA complexes in malaria. Nat. Commun..

[29] Regev-Rudzki N., Wilson D.W., Carvalho T.G., Sisquella X., Coleman B.M., Rug M., Bursac D., Angrisano F., Gee M., Hill A.F. (2013). Cell-cell communication between malaria-infected red blood cells via exosome-like vesicles. Cell.

[30] Correa R., Coronado L., Caballero Z., Faral-Tello P., Robello C., Spadafora C. (2019). Extracellular vesicles carrying lactate dehydrogenase induce suicide in increased population density of *Plasmodium falciparum* in vitro. Sci. Rep..

[31] Pena R.T., Blasco L., Ambroa A., Gonzalez-Pedrajo B., Fernandez-Garcia L., Lopez M., Bleriot I., Bou G., Garcia-Contreras R., Wood T.K. (2019). Relationship between quorum sensing and secretion systems. Front. Microbiol..

[32] Briggs E.M., Rojas F., McCulloch R., Matthews K.R., Otto T.D. (2021). Single-cell transcriptomic analysis of bloodstream *Trypanosoma brucei* reconstructs cell cycle progression and developmental quorum sensing. Nat. Commun..

[33] Tettey M.D., Rojas F., Matthews K.R. (2022). Extracellular release of two peptidases dominates generation of the trypanosome quorum-sensing signal. Nat. Commun..

[34] Gualdron-Lopez M., Flannery E.L., Kangwanrangsan N., Chuenchob V., Fernandez-Orth D., Segui-Barber J., Royo F., Falcon-Perez J.M., Fernandez-Becerra C., Lacerda M.V.G. (2018). Characterization of *Plasmodium vivax* proteins in plasma-derived exosomes from malaria-infected liver-chimeric humanized mice. Front. Microbiol..

[35] Aparici-Herraiz I., Gualdron-Lopez M., Castro-Cavadia C.J., Carmona-Fonseca J., Yasnot M.F., Fernandez-Becerra C., Del Portillo H.A. (2021). Antigen discovery in circulating extracellular vesicles from *Plasmodium vivax* patients. Front. Cell Infect. Microbiol..

[36] Salzer U., Hinterdorfer P., Hunger U., Borken C., Prohaska R. (2002). Ca(++)-dependent vesicle release from erythrocytes involves stomatin-specific lipid rafts, synexin (annexin VII), and sorcin. Blood J. Am. Soc. Hematol..

[37] Lowry O.H., Rosebrough N.J., Farr A.L., Randall R.J. (1951). Protein measurement with the Folin phenol reagent. J. Biol. Chem..

[38] Howe E.A., Sinha R., Schlauch D., Quackenbush J. (2011). RNA-Seq analysis in MeV. Bioinformatics.

[39] Bardou P., Mariette J., Escudie F., Djemiel C., Klopp C. (2014). jvenn: An interactive Venn diagram viewer. BMC Bioinform..

[40] Mi H., Muruganujan A., Ebert D., Huang X., Thomas P.D. (2019). PANTHER version 14: More genomes, a new PANTHER GO-slim and improvements in enrichment analysis tools. Nucleic Acids Res..

[41] Szklarczyk D., Santos A., von Mering C., Jensen L.J., Bork P., Kuhn M. (2016). STITCH 5: Augmenting protein-chemical interaction networks with tissue and affinity data. Nucleic Acids Res..

[42] Fonseka P., Pathan M., Chitti S.V., Kang T., Mathivanan S. (2021). FunRich enables enrichment analysis of OMICs datasets. J. Mol. Biol..

[43] Rivadeneira E.M., Wasserman M., Espinal C.T. (1983). Separation and concentration of schizonts of *Plasmodium falciparum* by Percoll gradients. J. Protozool..

[44] Vimonpatranon S., Chotivanich K., Sukapirom K., Lertjuthaporn S., Khowawisetsut L., Pattanapanyasat K. (2019). Enumeration of the invasion efficiency of *Plasmodium falciparum* in vitro in four different red blood cell populations using a three-color flow cytometry-based method. Cytom. Part A.

[45] Thery C., Witwer K.W., Aikawa E., Alcaraz M.J., Anderson J.D., Andriantsitohaina R., Antoniou A., Arab T., Archer F., Atkin-Smith G.K. (2018). Minimal information for studies of extracellular vesicles 2018 (MISEV2018): A position statement of the International Society for Extracellular Vesicles and update of the MISEV2014 guidelines. J. Extracell Vesicles.

[46] Bryk A.H., Wisniewski J.R. (2017). Quantitative Analysis of Human Red Blood Cell Proteome. J. Proteome Res..

[47] Ravenhill B.J., Kanjee U., Ahouidi A., Nobre L., Williamson J., Goldberg J.M., Antrobus R., Dieye T., Duraisingh M.T., Weekes M.P. (2019). Quantitative comparative analysis of human erythrocyte surface proteins between individuals from two genetically distinct populations. Commun. Biol..

[48] Keerthikumar S., Chisanga D., Ariyaratne D., Al Saffar H., Anand S., Zhao K., Samuel M., Pathan M., Jois M., Chilamkurti N. (2016). ExoCarta: A Web-Based Compendium of Exosomal Cargo. J. Mol. Biol..

[49] Kuhn M., von Mering C., Campillos M., Jensen L.J., Bork P. (2008). STITCH: Interaction networks of chemicals and proteins. Nucleic Acids Res..

[50] Dekel E., Yaffe D., Rosenhek-Goldian I., Ben-Nissan G., Ofir-Birin Y., Morandi M.I., Ziv T., Sisquella X., Pimentel M.A., Nebl T. (2021). 20S proteasomes secreted by the malaria parasite promote its growth. Nat. Commun..

[51] Yoon Y.J., Kim O.Y., Gho Y.S. (2014). Extracellular vesicles as emerging intercellular communicasomes. BMB Rep..

[52] Opadokun T., Rohrbach P. (2021). Extracellular vesicles in malaria: An agglomeration of two decades of research. Malar. J..

[53] Sampaio N.G., Cheng L., Eriksson E.M. (2017). The role of extracellular vesicles in malaria biology and pathogenesis. Malar. J..

[54] Antwi-Baffour S., Adjei J.K., Agyemang-Yeboah F., Annani-Akollor M., Kyeremeh R., Asare G.A., Gyan B. (2016). Proteomic analysis of microparticles isolated from malaria positive blood samples. Proteome Sci..

[55] Martin-Jaular L., Nakayasu E.S., Ferrer M., Almeida I.C., Del Portillo H.A. (2011). Exosomes from *Plasmodium yoelii*-infected reticulocytes protect mice from lethal infections. PLoS ONE.

[56] Abdi A., Yu L., Goulding D., Rono M.K., Bejon P., Choudhary J., Rayner J. (2017). Proteomic analysis of extracellular vesicles from a *Plasmodium falciparum* Kenyan clinical isolate defines a core parasite secretome. Wellcome Open Res..

[57] Ponnudurai T., Leeuwenberg A.D., Meuwissen J.H. (1981). Chloroquine sensitivity of isolates of *Plasmodium falciparum* adapted to in vitro culture. Trop. Geogr. Med..

[58] Bryant J.M., Baumgarten S., Lorthiois A., Scheidig-Benatar C., Claes A., Scherf A. (2018). De Novo Genome Assembly of a *Plasmodium falciparum* NF54 clone using single-molecule real-time sequencing. Genome Announc..

[59] Delves M.J., Straschil U., Ruecker A., Miguel-Blanco C., Marques S., Dufour A.C., Baum J., Sinden R.E. (2016). Routine in vitro culture of *P. falciparum* gametocytes to evaluate novel transmission-blocking interventions. Nat. Protoc..

[60] Prudent M., Delobel J., Hubner A., Benay C., Lion N., Tissot J.D. (2018). Proteomics of stored red blood cell membrane and storage-induced microvesicles reveals the association of flotillin-2 with band 3 complexes. Front. Physiol..

[61] Chiangjong W., Netsirisawan P., Hongeng S., Chutipongtanate S. (2021). Red blood cell extracellular vesicle-based drug delivery: Challenges and opportunities. Front. Med..

[62] Thangaraju K., Neerukonda S.N., Katneni U., Buehler P.W. (2020). Extracellular vesicles from red blood cells and their evolving roles in health, coagulopathy and therapy. Int. J. Mol. Sci..

[63] Tiberti N., Latham S.L., Bush S., Cohen A., Opoka R.O., John C.C., Juillard A., Grau G.E., Combes V. (2016). Exploring experimental cerebral malaria pathogenesis through the characterisation of host-derived plasma microparticle protein content. Sci. Rep..

[64] Avalos-Padilla Y., Georgiev V.N., Lantero E., Pujals S., Verhoef R., Borgheti-Cardoso L.N., Albertazzi L., Dimova R., Fernandez-Busquets X. (2021). The ESCRT-III machinery participates in the production of extracellular vesicles and protein export during *Plasmodium falciparum* infection. PLoS Pathog..

[65] Simpson J.A., Silamut K., Chotivanich K., Pukrittayakamee S., White N.J. (1999). Red cell selectivity in malaria: A study of multiple-infected erythrocytes. Trans. R. Soc. Trop. Med. Hyg..

[66] Chotivanich K., Udomsangpetch R., Simpson J.A., Newton P., Pukrittayakamee S., Looareesuwan S., White N.J. (2000). Parasite multiplication potential and the severity of Falciparum malaria. J. Infect. Dis..

