# Supplementary material for: Extracellular Vesicles Derived from Early and Late Stage Plasmodium falciparum-Infected Red Blood Cells Contain Invasion-Associated Proteins
Source: J Clin Med. 2022 Jul 21;11(14):4250. doi: 10.3390/jcm11144250 (PMC9318397; doi:10.3390/jcm11144250)
Supplement: Supplementary file 1 [file jcm-11-04250-s001.zip › jcm-1799635-supplementary.pdf]

Supplementary Materials

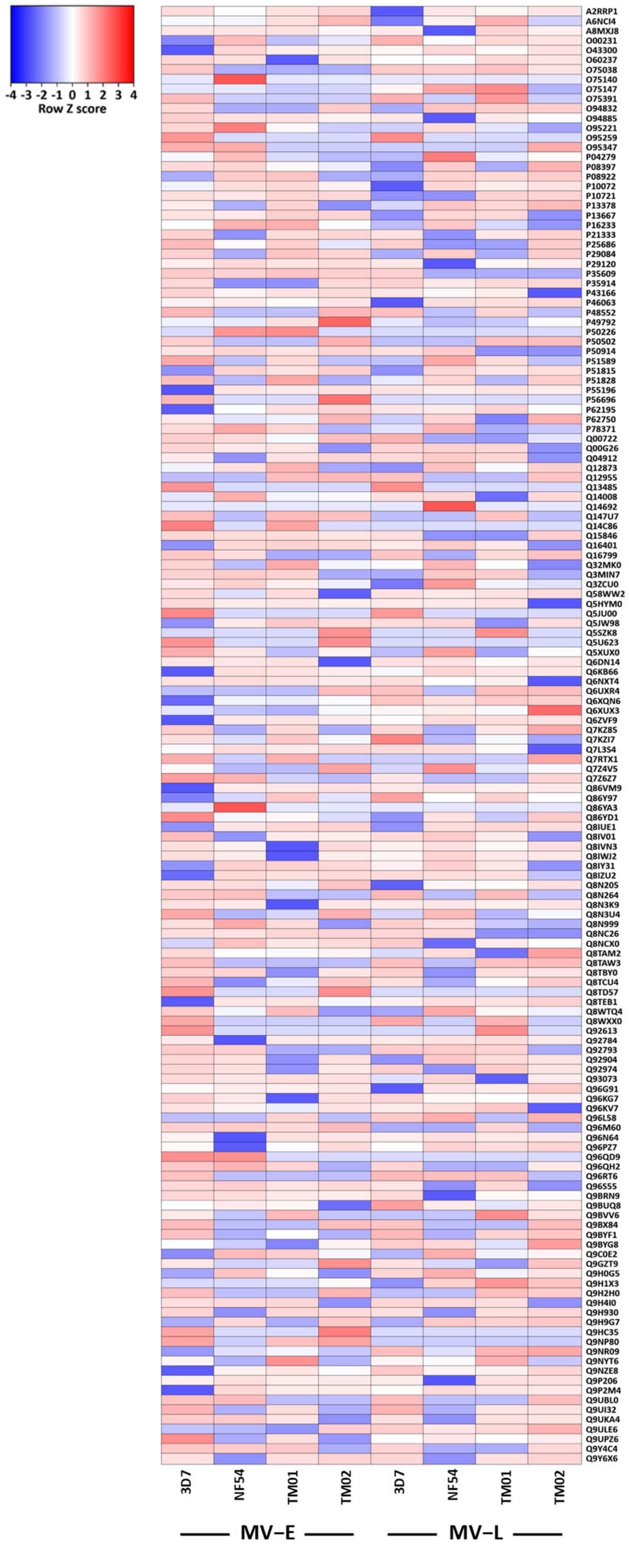

Figure S1 Heatmap of unique human proteins carried in *Pf*-MV. Side bar indicates differential protein expression between each sample (blue: decrease, red: increase). Rows: proteins; columns: samples.

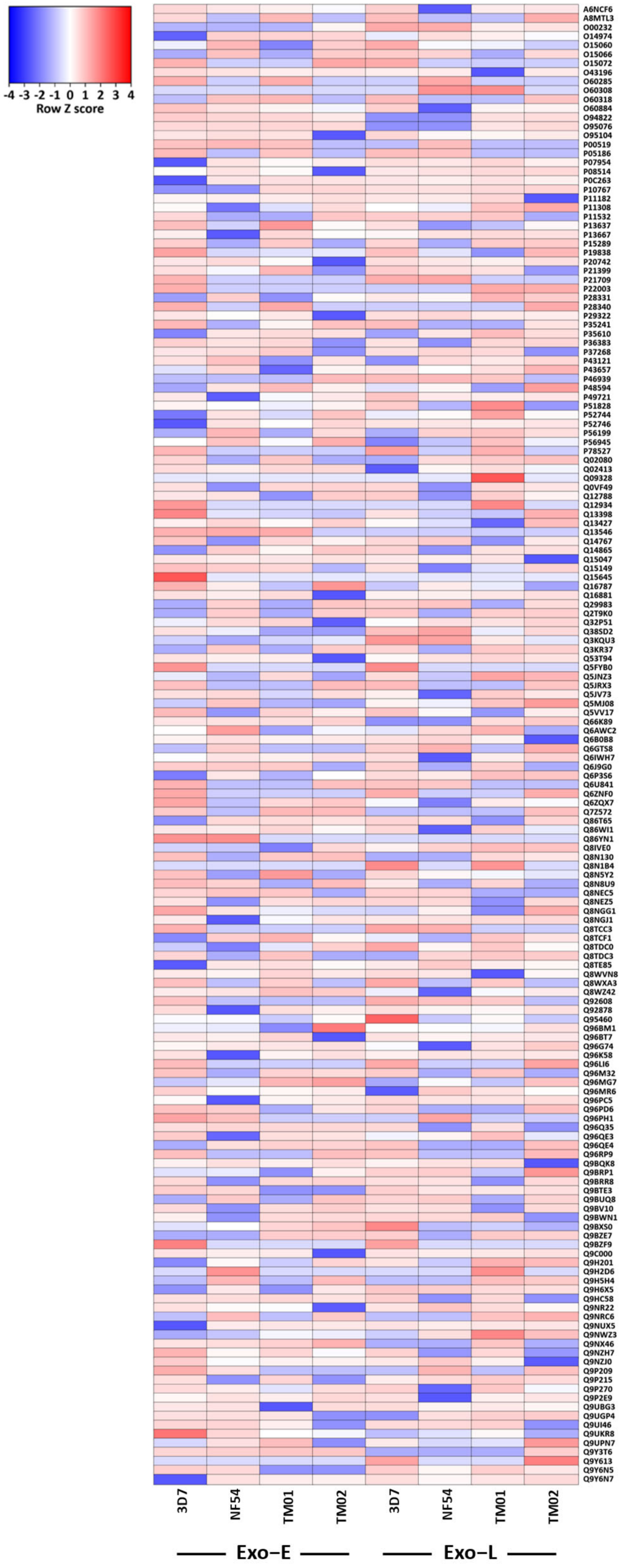

Figure S2 Heatmap of unique human proteins carried in *Pf*-Exo. Side bar indicates differential protein expression between each sample (blue: decrease, red: increase). Rows: proteins; columns: samples.

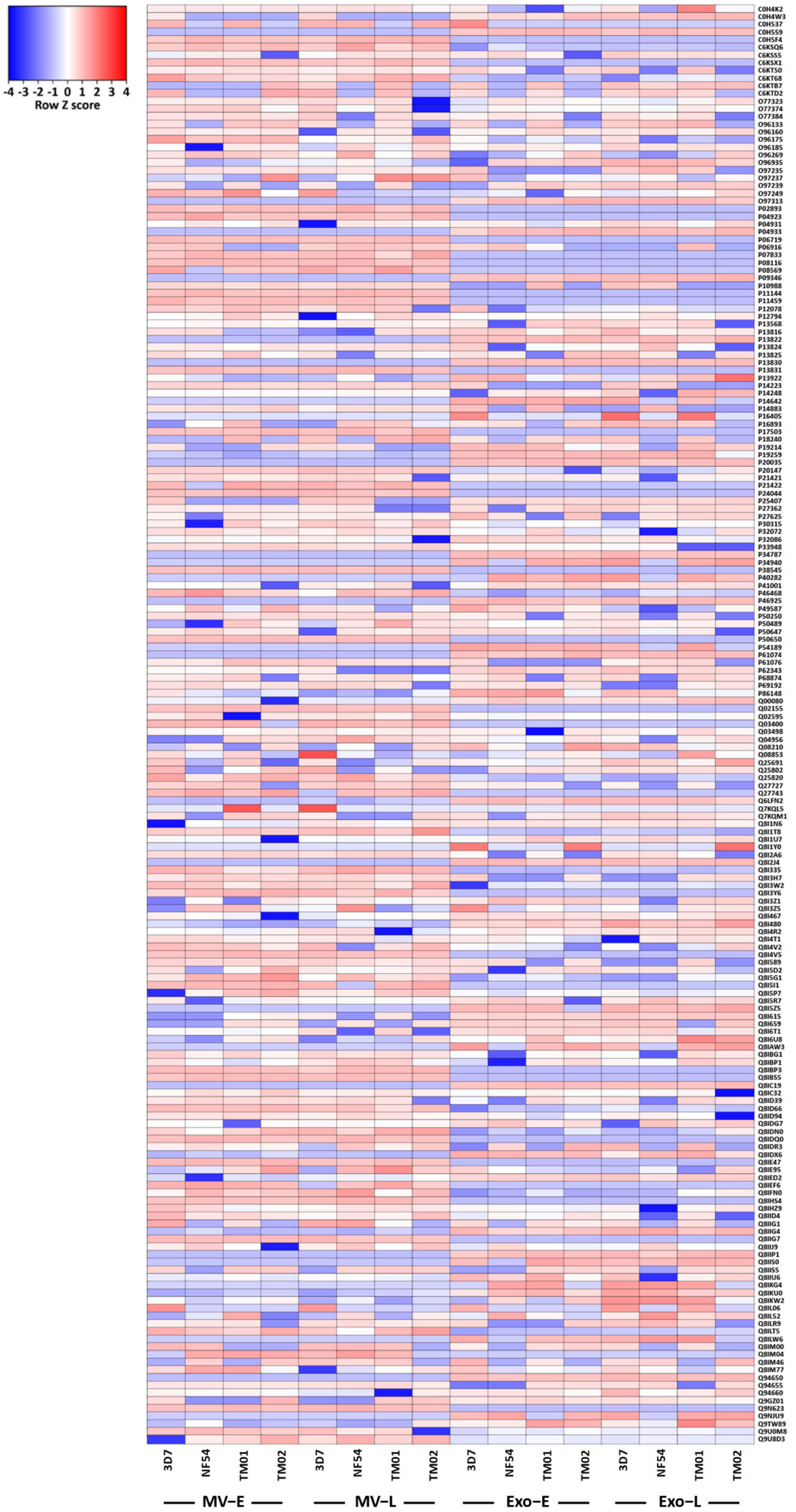

Figure S3 Heatmap analysis of differential expression levels of *P. falciparum* proteins in each type of *Pf*-EVs from 4 *P. falciparum* strains. Side bar indicates differential protein expression between each sample (blue: decrease, red: increase). Rows: proteins; columns: samples.
